# Decreased mitochondrial electron transport proteins and increased complement mediators in plasma neural-derived exosomes of early psychosis

**DOI:** 10.1038/s41398-020-01046-3

**Published:** 2020-10-26

**Authors:** Edward J. Goetzl, Vinod H. Srihari, Sinan Guloksuz, Maria Ferrara, Cenk Tek, George R. Heninger

**Affiliations:** 1 Department of Medicine, University of California Medical Center, San Francisco, CA USA; 2 Department of Psychiatry, Yale University School of Medicine and Connecticut Mental Health Center, New Haven, CT USA

**Keywords:** Prognostic markers, Molecular neuroscience

## Abstract

Potentially neurotoxic systems involved in traumatic and degenerative diseases of the brain were assessed in acute psychosis. Astrocyte-derived exosomes (ADEs) and neuron-derived exosomes (NDEs) were immunoprecipitated from plasma of ten untreated first-episode psychotics (FPs) and ten matched normal controls (Cs). Neural mitochondrial electron transport and complement proteins were extracted, quantified by ELISAs and normalized with levels of CD81 exosome marker. Levels of subunits 1 and 6 of NADH-ubiquinone oxidoreductase (complex I) and subunit 10 of cytochrome b-c1 oxidase (complex III), but not of subunit 1 of cytochrome C oxidase (complex IV) or superoxide dismutase 1 (SOD1) were significantly lower in ADEs and NDEs of FPs than Cs. This dysregulated pattern of electron transport proteins is associated with increased generation of reactive oxygen species. ADE glial fibrillary acidic protein levels were significantly higher in FPs than Cs, indicating a higher percentage of inflammatory astrocytes in FPs. ADE levels of C3b opsonin were significantly higher and those of C5b-9 attack complex was marginally higher in FPs than Cs. A significantly lower ADE level of the C3 convertase inhibitor CD55 may explain the higher levels of C3 convertase-generated C3b. ADE levels of the neuroprotective protein leukemia inhibitory factor (LIF) were significantly lower in FPs than Cs, whereas levels of IL-6 were no different. Plasma neural exosome levels of electron transport and complement proteins may be useful in predicting FP and guiding therapy. SOD mimetics, C3 convertase inhibitors and LIF receptor agonists also may have therapeutic benefits in FP.

## Introduction

Most studies of the neurobiology of schizophrenia have focused on neurotransmitter systems, their receptors, and downstream effectors. In the past decade, however, many findings of astroglial cell abnormalities in human brain tissues from schizophrenia patients, involving their numbers, gene expression, neuromediator metabolism and interactions with neurons have suggested a central role in pathogenesis^1–5^. Astrocytes are abundant glial cells of the central nervous system that normally serve diverse trophic roles for neurons^6^. In many inflammatory and degenerative neurological diseases, however, astrocytes increase in number, are transformed into A1 reactive/inflammatory-type astrocytes and contribute to destruction of neurons^7–9^. The mechanisms by which inflammatory-type astrocytes damage neurons in diseases have not been elucidated fully. Astrocytes and other neural cells secrete extracellular vesicles termed exosomes that contain proteins, nucleic acids and lipids representative of the cells of origin and reflective of physiological and pathological changes in these cells^10^. The recently developed capacity to specifically enrich astrocyte-derived exosomes (ADEs) from plasma of living subjects has enabled investigations into the roles of inflammatory astrocytes in Alzheimer’s disease (AD) and traumatic brain injury (TBI)^11,12^.

Among the most functionally-prominent constituents of ADEs from inflammatory-type astrocytes are proteins of the complement systems. Of the many complement components, the most damaging to neurons are C3b and C5b-9 terminal attack complex. C3b coats neurons and thereby facilitates high-affinity attachment of phagocytic microglia and their consequent neurotoxicity. C5b-9 attaches to and attacks neuronal plasma membranes with a direct neurotoxic outcome. In Mild Cognitive Impairment (MCI) that progresses to AD dementia within three years and in early clinically evident AD, exosome marker CD81-normalized ADE levels of complement effector components of the classical and alternative pathways including C3b and C5b-9 were higher than in matched cognitively normal controls^11,13^. Further, ADE levels of several complement-regulatory membrane proteins were already lower than in matched cognitively normal controls 5–12 years before onset of dementia. In sports-related TBI, CD81-normalized ADE levels of complement effector components of the classical, alternative and lectin pathways were higher than in matched cognitively normal controls acutely and for months^12^. The same pattern of elevated CD81-normalized ADE levels of complement effector components was detected in TBI of military veterans and some persisted for years^12^. In TBI of both populations, the ADE levels of complement components including C3b and C5b-9 were 12- to 35-fold higher than those in neuron-derived exosomes.

We therefore enriched plasma ADEs and neuron-derived exosomes (NDEs) from patients with acute first-episode psychosis (FP) and from matched controls for analyses of complement and mitochondrial electron transport proteins. Our data show diverse abnormalities in FP patients, including elevated ADE levels of neurotoxic complement proteins as well as dysfunctional NDE and ADE levels of electron transport system proteins, that consequently would produce higher levels of reactive oxygen species (ROS) and lower quantities of ATP.

## Methods

### Research participant selection and evaluation

Subjects were recruited from a specialized treatment early in psychosis (STEP) program of the Connecticut Mental Health Center (New Haven, CT). All had suffered onset of psychosis within two years before recruitment. Excluded were those patients and controls with affective psychosis, psychosis secondary to substance use or medical illness, pregnancy or current breast-feeding, physical or laboratory evidence of inflammation or exposure to any drug affecting inflammation in the prior four weeks. Also excluded were those unable to communicate in English or with co-morbid unstable serious medical illness. The study was approved by the Yale University Medical Center Institutional Review Group for Human Research and each participant signed a consent form.

On admission, all participants were assessed for severity of psychosis and mood symptoms, neurocognitive function, and premorbid life state and adjustments, including the total (score 30–210) and general psychopathology (score 16–112) positive and negative syndrome scales (PANSS)^14^. Ten consecutive first-episode psychotics (FPs) with a duration of untreated psychosis shorter than two years were selected for this study (Table 1)^15^. One subject had DSM IV TR 295.1 disorganized schizophrenia, three subjects had DSM IV TR 295.3 schizophrenia paranoid type, one subject had DSM IV TR 295.4 schizophreniform disorder, and five subjects had DSM IV TR 295.9 undifferentiated schizophrenia. Plasmas for exosome studies were collected in the morning both for controls and for FP patients after the day of admission and stored at −80 °C.

**Table 1:** Participant Characteristics.

Group	Age,years (mean ± S.D.)	Male Female	Days from onset to admission (mean,range)	PANSS,Total (mean ± S.D.)	PANSS, General Psychopathology (mean ± S.D.)
Controls (*n* = 10)	22.8 ± 3.44	7 3			
First Episode Psychotics (*n* = 10)	21.5 ± 3.28	7 3	113, 14–338	98.2 ± 17.0	63.4 ± 13.4

*PANSS* positive and negative symptom scales.

### Enrichment of plasma neuron-derived and astrocyte-derived extracellular vesicles

Aliquots of 0.25 mL plasma were incubated with 0.1 mL of thromboplastin D (Thermo Fisher Scientific, Waltham, MA) for 30 min at room temperature, followed by addition of 0.15 mL of calcium- and magnesium-free Dulbecco’s balanced salt solution (DBS) with protease inhibitor cocktail (Roche, Indianapolis, IN) and phosphatase inhibitor cocktail (Thermo Fisher Scientific; DBS + + ) as described^10,11^. After centrifugation at 3000 × *g* for 30 min at 4 °C, total exosomes were harvested from resultant supernatants by precipitation with 126 μL per tube of ExoQuick (System Biosciences, Mountain View, CA) and centrifugation at 1500 × *g* for 30 min at 4 °C. To separately enrich neuron-derived exosomes (NDEs) and astrocyte-derived exosomes (ADEs), replicate preparations of total exosomes were resuspended in 0.35 mL of DBS and incubated for 60 min at room temperature with either 2.0 μg of mouse anti-human CD171 (L1CAM neural adhesion protein) biotinylated antibody (clone 5G3; eBiosciences, San Diego, CA) or mouse anti-human glutamine aspartate transporter (GLAST, ACSA-1) biotinylated antibody (Miltenyi Biotec, Auburn, CA), respectively, in 50 μL of 3% bovine serum albumin (BSA; 1:3.33 dilution of Blocker BSA 10% solution in DBS; Thermo Fisher Scientific) per tube with mixing, followed by addition of 10 μL of streptavidin agarose Ultralink resin (Thermo Fisher Scientific) in 40 μL of 3% BSA and incubation for 30 minutes at room temperature with mixing. After centrifugation at 800 × *g* for 10 min at 4 °C and removal of the supernatant, each pellet was suspended in 100 μL of cold 0.05 M glycine-HCl (pH 3.0) by gentle mixing for 10 s and centrifuged at 4000 × *g* for 10 min, all at 4 °C. Supernatants then were transferred to clean tubes containing 25 μL of 10% BSA and 10 μL of 1 M Tris–HCl (pH 8.0) and mixed gently. An aliquot of 5 μL was removed from each tube for EV counts before addition of 370 μL of mammalian protein extraction reagent (M-PER, Thermo Fisher Scientific). Resultant 0.5 mL lysates of NDEs and ADEs were stored at −80 °C.

For counting and sizing of exosomes, each suspension was diluted 1:50 in DBS. The mean diameter (nanometers) and concentration (particles per milliliter) of exosomes in each suspension were determined by nanoparticle tracking analysis (NTA) using the Nanosight NS500 system with a G532nm laser module and NTA 3.1 nanoparticle tracking software (Malvern Instruments, Malvern, United Kingdom). Camera settings were as follows: gain 366; shutter 31.48; and frame rate 24.9825 frames/s. Brownian motion was captured by performing 5 repeated 60 s video recordings.

### Quantification of NDE and ADE proteins

ADE and NDE proteins were quantified by enzyme-linked immunosorbent assay (ELISA) kits for human tetraspanning exosome marker CD81, subunit 6 of NADH-ubiquinone oxidoreductase (complex I), decay-accelerating factor (CD55) (Cusabio Technology by American Research Products, Waltham, MA), glutamine synthetase, subunit 1 of cytochrome C oxidase (complex IV) (Cloud-Clone Corp by American Research Products), subunit 1 of NADH-ubiquinone oxidoreductase (complex I) (DL-Develop Corp. by American Research Products), subunit 10 of cytochrome b-c1 oxidase (complex III) (Abbkine, Inc. by American Research Products), glial fibrillary acidic protein (Millipore-Sigma Corp., Burlington, MA), complement fragment C3b (Abcam, Cambridge, MA), TCC C5b-9 (Aviva Systems, San Diego, CA), superoxide dismutase 1 (SOD1), CD59 (Ray Biotech, Norcross, GA), IL-6, neuron-specific enolase (R&D Systems-Bio-Techne, Minneapolis, MN), and leukemia inhibitory factor (LIF) (Thermo Fisher Scientific, Waltham,MA). ELISAs were conducted with blinding of researcher to sources of plasmas.

The mean value for all determinations of CD81 in each assay group was set at 1.00, and relative values of CD81 for each sample were used to normalize their recovery.

## Results

### Participant characteristics

Demographics of controls matched those of the first-episode psychotics (FPs) (Table 1). The severity of psychosis was moderate to severe based on evaluation of total and general psychopathology PANSS.

### Exosome analyses

**Fig. 1:**
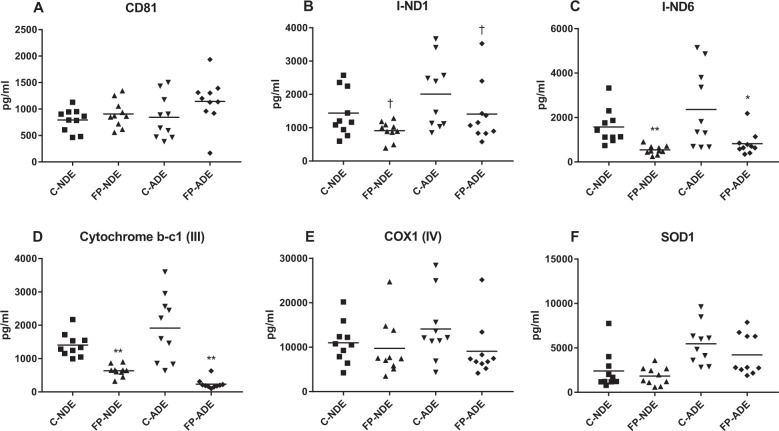
Protein constituents of the mitochondrial oxidative phosphorylation system in NDEs and ADEs. Each point represents the value for one study participant. The mean ± S.E.M. of control (C) and first-episode psychosis (FP) groups, respectively, were 792 ± 68.2 pg/ml and 907 ± 80.4 pg/ml for NDEs and 846 ± 129 pg/ml and 1146 ± 141 pg/ml for ADEs for CD81 (**a**); 1437 ± 223 pg/ml and 912 ± 89.2 pg/ml for NDEs and 2010 ± 326 pg/ml and 1195 ± 161 pg/ml for ADEs for ND1 subunit of NADH-ubiquinone oxidoreductase (complex I) (**b**); 1574 ± 246 pg/ml and 542 ± 62.1 pg/ml for NDEs and 2363 ± 458 pg/ml and 825 ± 166 pg/ml for ADEs for ND6 subunit of NADH-ubiquinone oxidoreductase (complex I) (**c**); 1404 ± 112 pg/ml and 635 ± 53.8 pg/ml for NDEs and 1916 ± 315 pg/ml and 236 ± 47.2 pg/ml for ADEs for subunit 10 of cytochrome b-c1 (complex III) (**d**); 10975 ± 1461 pg/ml and 9750 ± 2011 pg/ml for NDEs and 14080 ± 2348 pg/ml and 9080 ± 1948 pg/ml for ADEs for subunit 1 of cytochrome c oxidase (complex IV) (**e**); and 2403 ± 672 pg/ml and 1829 ± 315 pg/ml for NDEs and 5472 ± 724 pg/ml and 4219 ± 723 pg/ml for ADEs for superoxide dismutase 1 (SOD1) (**f**). All values in B – F and in Fig. 2 were normalized for content of the exosome marker CD81. Statistical significance of differences in values between C and FP groups for NDEs and ADEs were calculated by two sample t tests; ^†^, *p* < 0.05; *, *p* < 0.01; **, *p* < 0.001.

Mean ± S.E.M. counts of ADEs in immuno-selected suspensions resembled those of past studies at 60.9 ± 2.43 ×10^9^/ml for controls and 61.3 ± 2.80 ×10^9^/ml for FP patients. Counts of NDEs were 134 ± 3.92 ×10^9^/ml for controls and 135 ± 5.69 × 10^9^/ml for FP patients. CD81 exosome marker levels in the ADE and NDE populations also showed no significant differences between those of controls and FP patients (Fig. 1a). ADEs and NDEs of controls and FP patients were of similar sizes with diameters ranging from 64 to 120 nm for NDEs and 72–114 nm for ADEs. Levels of markers representative of each type of extracellular vesicle in initial pilot preparations confirmed a high degree of enrichment. The neuron marker neuron-specific enolase was at a mean ± S.E.M. of 5,816 ± 142 pg/ml and 5,238 ± 160 pg/ml in control and FP patient NDE extracts, respectively, and 290 ± 31.4 pg/ml and 352 ± 41.8 pg/ml in control and FP patient ADE extracts. The astrocyte marker glial fibrillary acidic protein (GFAP) was at a mean ± S.E.M. of 111,614 ± 14,822 pg/ml and 468,264 ± 51,590 pg/ml in control and FP patient ADE extracts, respectively, and 4,958 ± 592 pg/ml and 5,326 ± 755 pg/ml in control and FP patient NDE extracts.

### Mitochondrial electron transport system proteins

CD81-normalized ADE and NDE levels of subunit 1 of NADH-ubiquinone oxidoreductase (complex I) were marginally significantly lower in FP patients than controls, whereas those of the more catalytically important subunit 6 of NADH-ubiquinone oxidoreductase were significantly lower in FP patients than controls (Fig. 1b, c). CD81-normalized ADE and NDE levels of cytochrome b-c1 oxidase (coenzyme Q-cytochrome C oxidoreductase) (complex III) also were significantly lower in FP patients than controls (Fig. 1d). In contrast, CD81-normalized ADE and NDE levels of cytochrome C oxidase 1 (complex IV) were no different in FP patients than controls (Fig. 1e). This pattern of depressed levels of early mitochondrial electron transport complexes in the presence of a normal level of the terminal complex IV has been associated with increased generation of reactive oxygen species^16^.

CD81-normalized ADE and NDE levels of superoxide dismutase 1 (SOD1), which is the predominant scavenger of mitochondrially-produced superoxide anion, were statistically the same in FP patients and controls (Fig. 1f).

### Exosome complement and cytokine constituents

**Fig. 2:**
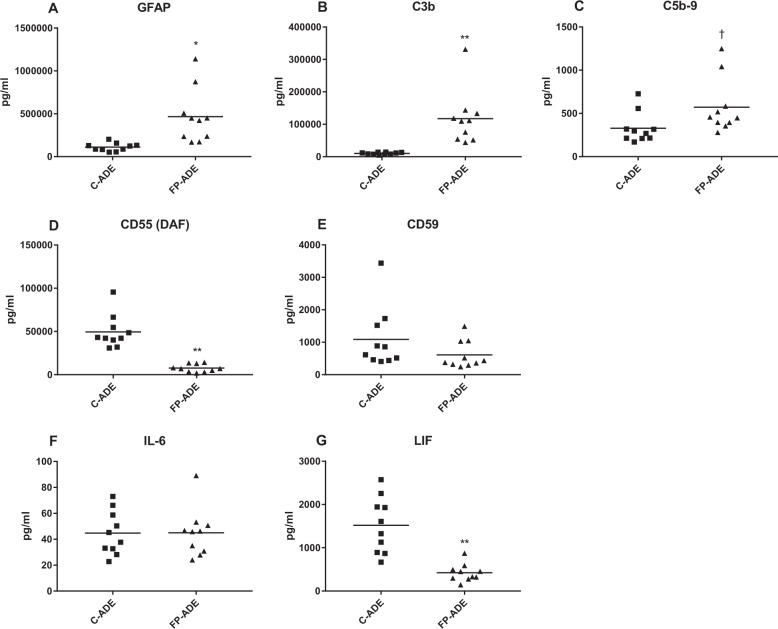
Complement and cytokine effector proteins in ADEs. Each point represents the value for one study participant. The mean ± S.E.M. of control (C) and first-episode psychosis (FP) groups, respectively, were 111,641 ± 14,822 pg/ml and 466,335 ± 100,239 pg/ml for GFAP (**a**); 10,167 ± 994 pg/ml and 117,468 ± 26,305 pg/ml for C3b (**b**); 330 ± 56.0 pg/ml and 573 ± 100 pg/ml for C5b-9 (**c**); 49,621 ± 6073 pg/ml and 7652 ± 1452 pg/ml for CD55 (DAF) (**d**); 1087 ± 299 pg/ml and 613 ± 134 pg/ml for CD59 (**e**); 44.8 ± 5.35 pg/ml and 45.0 ± 5.85 pg/ml for IL-6 (**f**); and 1519 + 204 pg/ml and 424 + 64.2 pg/ml for LIF (**g**). Statistical significance of differences in values between C and FP groups were calculated by two sample t tests; ^†^, *p* < 0.05; *, *p* < 0.01; **, *p* < 0.001.

CD81-normalized ADE levels of GFAP, a major biomarker of inflammatory-type astrocytes, were significantly higher in FP patients than controls (Fig. 2a). As ADE counts and levels of the exosome marker CD81 were no different in FP patients than controls, the much higher levels of ADE GFAP in FP patients than controls is indicative of a higher proportion of activated inflammatory astrocytes in FP patients. Inflammatory-type astrocytes are the source of inflammatory neurotoxic complement mediators and their levels in ADEs reflect those in such astrocytes^11,12,17^. ADE levels of neuron-opsonizing C3b were significantly higher in FP patients than controls (Fig. 2b), which is evidence of greater activation of astrocyte complement pathways in FP patients than controls. However, ADE levels of the directly neurotoxic C5b-9 attack complex were only marginally higher in FP patients than controls (Fig. 2c). This dissociation is at least partially explained by the ADE levels of membrane-associated complement regulatory proteins CD55 and CD59. CD55 levels were significantly lower in FP patients compared to those in controls, which would allow for higher generation of the C3b product by C3 convertases of both the classical and alternative pathways (Fig. 2d). The normal CD59 level of ADEs in FP patients relative to those in controls, however, would suppress formation of C5b-9 from C3b (Fig. 2e).

Astrocyte-derived leukemia inhibitory factor (LIF), a major neuroprotective cytokine^18^, was detected at significantly lower levels in ADEs of FP patients than controls (Fig. 2g). In contrast, the predominantly microglial-derived inflammatory cytokine IL-6, that is high in plasma and brain tissues in many neural diseases, was present at only very low levels in ADEs with no difference between FP patients and controls (Fig. 2f).

## Discussion

High levels of mitochondrial-derived reactive oxygen species (ROS), composed predominantly of superoxide anion (O_2_^−^) with lesser amounts of hydroperoxide and hydroxyl radical, damage neurons by nucleic acid oxidation and lipid peroxidation. Results of several types of proteomic studies of mitochondrial electron transport proteins previously revealed that the earliest abnormalities in Alzheimer’s disease (AD) were reduced levels of native complexes I and III^16^. In contrast, decreases in the level of complex IV that are typical of normal aging and greater in AD occurred later in the course concurrent with decline in synaptic connectivity^19,20^. The pattern of decreases in levels of complexes I and III, while that of complex IV remains normal, results in increased generation of ROS. Less is known about changes in the mitochondrial electron transport system in acute psychosis and schizophrenia, but especially prominent among pathogenically-related decreased levels of DNA and transcriptional activity in schizophrenia are decreases in complex I genes, proteins and activities^21,22^. The significant decreases in levels of complex I protein in brain tissues of schizophrenic patients have been attributed partially to medications^23^. Nonetheless, induced pluripotent stem cell (iPSC) lines of interneurons from schizophrenic patients showed altered expression of genes encoding proteins involved in numerous mitochondrial activities, including increased generation of ROS^24^. Further there is extensive evidence of increased ROS in schizophrenia and animal models of schizophrenia^25,26^. Our novel findings of significantly reduced levels of major components of complexes I and III, but not of complex IV, strongly support prominently increased generation of ROS in FP (Fig. 1).

In neurodegenerative diseases, such as AD, and traumatic brain injury (TBI), ADE levels of the neurotoxic complement mediators C3b and C5b-9 are elevated relative to matched controls in part as a result of acquired concomitant deficiencies of complement regulatory membrane proteins CD55, CD59, and others^11,12^. In AD, where ADE complement effector levels are elevated and in elderly control subjects with much lower ADE levels of C3b and C5b-9, mean levels of C5b-9 were 1.49% and 2.68% of those of C3b, respectively. Similarly, in sports-related TBI, where ADE complement effector levels are elevated and in young control subjects with much lower ADE levels of C3b and C5b-9, mean levels of C5b-9 were 2.36% and 1.42% of those of C3b, respectively. The current unique findings in acute FP of a decreased ADE level of the C3 convertase inhibitor CD55 but a normal ADE level of CD59, that normally suppresses formation of C5b-9, leads to a greater elevation of C3b than C5b-9 relative to respective control levels (Fig. 2) and a low mean C5b-9 of 0.49% of that of C3b. Thus the direct neurotoxic activity of C5b-9 may contribute less than the opsonizing activity of C3b for microglial neurotoxicity in FP. Although there were no significant relationships between ADE levels of GFAP, C3b, and C5b-9, at least one of these levels was out of the control range for every FP patient. It was previously found that some alleles of C4 are associated with schizophrenia and with proportionate elevations of C4A in distinct regions of the brain^27,28^. Any relationship between our current findings and the apparent increase in susceptibility to schizophrenia attributable to C4 expression remain to be elucidated.

The complement-mediated neuroinflammatory and mitochondrial oxidative responses characteristic of FP and schizophrenia may in turn evoke the universal integrated stress response (ISR) of eukaryotic cells^29^. By broadly suppressing protein synthesis and stimulating synthesis of a few proteins in a new transcriptional program, the ISR either re-establishes neural homeostasis or induces apoptosis of the damaged cells. One consequence of activation of the ISR is prevention of development of long-term memory and other cognitive functions dependent on new protein production. Although suppression of the ISR has been thus suggested as a possible treatment for diseases of cognitive loss, the essential role of the ISR in numerous basic cellular adaptations indicate that therapeutic elimination of cellular stresses may be a more beneficial approach.

The present results suggest possible therapeutic avenues in acute schizophrenia, including SOD1 mimetics to scavenge higher than normal levels of superoxide anion, other scavengers specific for peroxides and hydroxyl radical components of ROS, and CD55-like inhibitors of C3 convertases. The very low levels of the LIF neuroprotective factor in ADEs of acute FP patients indicate possible benefits from LIF supplements or other LIF receptor agonists, as has been recommended for several neurological diseases^18^.

## References

[1] Kerns D (2010). Gene expression abnormalities and oligodendrocyte deficits in the internal capsule in schizophrenia. Schizophr. Res..

[2] Katsel P (2011). Astrocyte and glutamate markers in the superficial, deep, and white matter layers of the anterior cingulate gyrus in schizophrenia. Neuropsychopharmacology.

[3] Bernstein HG, Steiner J, Guest PC, Dobrowolny H, Bogerts B (2015). Glial cells as key players in schizophrenia pathology: recent insights and concepts of therapy. Schizophr. Res.

[4] Mei YY, Wu DC, Zhou N (2018). Astrocytic regulation of glutamate transmission in schizophrenia. Front Psychiatry.

[5] Tarasov VV (2019). Alterations of astrocytes in the context of schizophrenic dementia. Front Pharm..

[6] Sofroniew MV, Vinters HV (2010). Astrocytes: biology and pathology. Acta Neuropathol..

[7] Choi SS, Lee HJ, Lim I, Satoh J, Kim SU (2014). Human astrocytes: secretome profiles of cytokines and chemokines. PLoS ONE.

[8] Ben Haim L, Carrillo-de Sauvage MA, Ceyzeriat K, Escartin C (2015). Elusive roles for reactive astrocytes in neurodegenerative diseases. Front Cell Neurosci..

[9] Liddelow SA, Barres BA (2017). Reactive astrocytes: production, function, and therapeutic potential. Immunity.

[10] Goetzl EJ (2016). Cargo proteins of plasma astrocyte-derived exosomes in Alzheimer’s disease. FASEB J..

[11] Goetzl EJ, Schwartz JB, Abner EL, Jicha GA, Kapogiannis D (2018). High complement levels in astrocyte-derived exosomes of Alzheimer disease. Ann. Neurol..

[12] Goetzl EJ (2020). Traumatic brain injury increases plasma astrocyte-derived exosome levels of neurotoxic complement proteins. Faseb J..

[13] Winston CN, Goetzl EJ, Schwartz JB, Elahi FM, Rissman RA (2019). Complement protein levels in plasma astrocyte-derived exosomes are abnormal in conversion from mild cognitive impairment to Alzheimer’s disease dementia. Alzheimers Dement.

[14] Srihari VH (2014). Reducing the duration of untreated psychosis and its impact in the U.S.: the STEP-ED study. BMC Psychiatry.

[15] Miller TJ (2003). Prodromal assessment with the structured interview for prodromal syndromes and the scale of prodromal symptoms: predictive validity, interrater reliability, and training to reliability. Schizophr. Bull..

[16] Adav SS, Park JE, Sze SK (2019). Quantitative profiling brain proteomes revealed mitochondrial dysfunction in Alzheimer’s disease. Mol. Brain.

[17] Liddelow SA (2017). Neurotoxic reactive astrocytes are induced by activated microglia. Nature.

[18] Davis SM, Pennypacker KR (2018). The role of the leukemia inhibitory factor receptor in neuroprotective signaling. Pharm. Ther..

[19] Selfridge JE, E L, Lu J, Swerdlow RH (2013). Role of mitochondrial homeostasis and dynamics in Alzheimer’s disease. Neurobiol. Dis..

[20] Kriebel M, Ebel J, Battke F, Griesbach S, Volkmer H (2020). Interference with complex IV as a model of age-related decline in synaptic connectivity. Front Mol. Neurosci..

[21] Duong A (2016). Regulators of mitochondrial complex I activity: a review of literature and evaluation in postmortem prefrontal cortex from patients with bipolar disorder. Psychiatry Res..

[22] Ben-Shachar D (2017). Mitochondrial multifaceted dysfunction in schizophrenia; complex I as a possible pathological target. Schizophr. Res..

[23] Rollins BL (2018). Mitochondrial complex I deficiency in schizophrenia and bipolar disorder and medication influence. Mol. Neuropsychiatry.

[24] Ni P (2020). iPSC-derived homogeneous populations of developing schizophrenia cortical interneurons have compromised mitochondrial function. Mol. Psychiatry.

[25] Do KQ, Cuenod M, Hensch TK (2015). Targeting oxidative stress and aberrant critical period plasticity in the developmental trajectory to schizophrenia. Schizophr. Bull..

[26] Steullet P (2017). Oxidative stress-driven parvalbumin interneuron impairment as a common mechanism in models of schizophrenia. Mol. Psychiatry.

[27] Sekar A (2016). Schizophrenia risk from complex variation of complement component 4. Nature.

[28] Woo JJ, Pouget JG, Zai CC, Kennedy JL (2020). The complement system in schizophrenia: where are we now and what’s next?. Mol. Psychiatry.

[29] Costa-Mattioli, M. & Walter, P. The integrated stress response: from mechanism to disease. *Science* **368**, 10.1126/science.aat5314 (2020). 10.1126/science.aat5314 PMC8997189 32327570

